# Chloroquine and sulphadoxine-pyrimethamine sensitivity of *Plasmodium falciparum *parasites in a Brazilian endemic area

**DOI:** 10.1186/1475-2875-8-156

**Published:** 2009-07-14

**Authors:** Bianca Ervatti Gama, Natália K Almeida de Oliveira, Mariano G Zalis, José Maria de Souza, Fátima Santos, Cláudio Tadeu Daniel-Ribeiro, Maria de Fátima Ferreira-da-Cruz

**Affiliations:** 1 Laboratory of Malaria Research, Instituto Oswaldo Cruz, Fiocruz, Rio de Janeiro (RJ), Brazil; 2 Laboratory of Infectiology and Parasitology, Hospital Universitário Clementino Fraga Filho, Federal University of Rio de Janeiro, Rio de Janeiro (RJ), Brazil; 3 Ambulatory and Laboratory of Malaria Clinical Assays, Secretariat of Health Vigilance, Instituto Evandro Chagas, Belém (PA), Brazil; 4 Laboratory of Entomology, LACEN, Porto Velho (RO), Brazil

## Abstract

**Background:**

The goal of the present study was the characterization of *Plasmodium falciparum *genes associated to malaria drug resistance (*pfcrt*, *pfdhfr *and *pfdhps)*, in samples from two Brazilian localities.

**Methods:**

Parasites from 65 *P. falciparum *samples were genotyped using nested-PCR and direct DNA sequencing.

**Results:**

Six resistant sulphadoxine-pyrimethamine (SP) *pfdhfr *genotypes and one haplotype associated to SP sensitivity were detected. For *pfcrt *gene, SVMNT chloroquine (CQ)-resistant genotype was detected as well as the CVMNK CQ-sensitive haplotype in the same sample from Paragominas, that showed a SP-sensitive genotype.

**Conclusion:**

This study is the first to document the sensitivity of *P. falciparum *parasites to CQ and SP in Brazilian field samples. The importance of these findings is discussed.

## Background

Malaria is a challenging infectious disease to many countries in the world, especially to those located in tropics and subtropical regions, and the increasing numbers of drug resistant parasites worsens this situation. In Brazil, as well as in other endemic countries, drug-sensitivity tests revealed that *Plasmodium falciparum *isolates displayed high levels of resistance to many drugs, including chloroquine (CQ) and sulphadoxine-pyrimethamine (SP) [1-4]. The first documented case of CQ resistance in Brazil was in 1954; in the sixties several authors in South America (SA) reported the occurrence of *in vivo *falciparum malaria resistance to CQ and amodiaquine. The spread of 4-amino-quinoleine resistant strains of *P. falciparum *lead also to the use of SP in the seventies. Unfortunately, at the end of eighteens the SP resistance it was no less than 90% [5] and at the beginning of nineties the CQ failure rate was considered 100% [6] and, consequently, the Brazilian National Malaria Programme withdrew SP and CQ for falciparum malaria treatment. Then, the combination quinine plus tetracycline was introduced, followed by the usage of the combination quinine plus doxycycline or, as second-line drug, mefloquine plus primaquine. In 2007, a fixed combination of artemether plus lumefantrine (Coartem^®^) was introduced as first-line drug [7] and since 2008, the fixed combination artesunate plus mefloquine (FarManguinhos, Fiocruz) was produced, and its implementation in Brazilian endemic areas is in progress to counteract parasite resistance, according to WHO guidelines [8].

Interestingly, the reemergence of CQ-sensitive *P. falciparum *parasites as well as the downturn of *P. falciparum *triple mutants associated to SP resistance, were reported after cessation of monotherapy using CQ or SP for the treatment of *P. falciparum *malaria [9-13]. These findings provide a rationale for the search of drug-sensitive haplotypes in *P. falciparum isolates *in Brazilian areas where the use of these two drugs for falciparum malaria treatment has been interrupted since 1990.

The goal at the present study was, therefore, the characterization of *pfcrt*, *pfdhfr *and *pfdhps *genes that are known molecular markers of *P. falciparum *resistance to CQ and SP [14].

## Methods

### Study sites, blood samples and DNA extraction

Parasites from 65 *P. falciparum *samples were genotyped. These samples were collected at the time of diagnosis from uncomplicated malaria patients living in two Brazilian malaria-endemic areas: Porto Velho – Rondônia state (n = 46), and Paragominas – Pará state (n = 19). After obtaining the informed consent, venous blood collection was done according to protocols previously approved by the ethics research committees of Fiocruz and of local study sites. DNA was extracted from 1 ml of cryopreserved blood using QIAamp midi columns as described by the manufacturer (Qiagen).

### Polymerase chain reactions (PCRs) and electrophoresis

A nested-PCR technique was employed in order to amplify a partial DNA sequence containing the major single-nucleotide polymorphisms (SNPs) related to drug-resistance for each target gene as: SNPs A16V/S, C50R, N51I, C59R, S108N, V140L plus I164L for *pfdhfr*, S436A/F/C, A437G, K540E, A581G plus A613T/S for *pfdhps*, and SNPs C72S, M74I, N75E/D plus K76T for *pfcrt*. The protocols of *pfdhfr *and *pfdhps *nested PCRs have been described elsewhere [15]. The amplification of the *pfcrt *gene fragment was performed with 5 μl of DNA into a 45 μl mixture containing 2.5 mM of MgCl_2_, 250 μM of dNTPs, 1.25 units of Amplitaq Gold^® ^DNA polymerase (Applied Biosystems), and 10 pmol of each primer (Alpha DNA): CRTP1 (5' CCG TTA ATA ATA AAT ACA CGC AG 3') and CRTP2 (5' CGG ATG TTA CAA AAC TAT AGT TAC C 3') to amplify a 537 bp region. The reactions were settled with an initial hold (95°C/10 minutes), 45 cycles (94°C/30 seconds, 56°C/30 seconds and 60°C/1 minute) plus one final hold (60°C/3 minutes). Then, 5 μl of the first PCR amplification were added to 45 μl of a second mixture containing the second set of primers (Alpha DNA): CRTD1 (5' TGT GCT CAT GTG TTT AAA CTT 3') and CRTD2 (5' CAA AAC TAT AGT TAC CAA TTT TG 3') and 32 steps (1 hold 95°C/10 min, 30 cycles 92°C/30 sec, 48°C/30 sec and 65°C/30 sec, and 1 hold 65°C/3 min) were performed to amplify a 134 bp fragment. PCR products were analysed by ethidium bromide-stained agarose-gel (2%) electrophoresis.

### DNA sequencing

After DNA purification using the Wizard SV Gel and PCR Clean-Up System (Promega), the amplified fragments were sequenced using Big Dye^® ^Terminator Cycle Sequencing Ready Reaction version 3.1 (Applied Biosystems) and ABI PRISM DNA Analyzer 3730 (Applied Biosystems) from the Genomic Platform/PDTIS/Fiocruz [16].

## Results

The *pfcrt *nested-PCR generated DNA fragments in all the 65 samples, the *pfdhps *in 63 samples and the *pfdhfr *nested-PCR in 52 samples, showing different sensitivity threshold among the PCRs.

The *pfdhfr *gene analysis revealed the existence of seven haplotypes: six associated with drug resistance profiles (ARICNVI, ACICNVI, ACICNVL, ACNRNVI, ACNCNVI and ACIRNVI) and one, from Paragominas locality, associated with SP sensitivity (ACNCSVI). The drug resistance *pfdhfr *haplotypes displayed up to three (50R + 51I and 108N; 51I + 108N and 164L, or 51I + 59R and 108N) out of seven SNPs herein analysed. Concerning the *pfdhps *gene, three single haplotypes (SGEGA, SGEAA, SGKAA), displaying up to three (437G + 540E and 581G) out of the five SNPs analysed were observed and, in this case, no drug sensitive haplotype (SAKAA) was detected. In relation to *pfcrt *gene, two single haplotypes were observed: the SVMNT CQ-resistant in 97% of the samples and the CVMNK CQ-sensitive in the same sample from Paragominas that showed the sensitive genotype to SP (*pfdhfr *gene). All the three genes displayed at least one mixed haplotype. These results are summarized in Table 1.

**Table 1:** *Pfcrt*, *pfdhfr *and *pfdhps *haplotypes of *P. falciparum *parasites from Paragominas (PRG) and Porto Velho (PV), Brazilian Amazon.

**Gene**	**Haplotypes**	**n (Locality)**	**%**	**Mutated codons**
***pfcrt***	SVMNT	63 (18 PRG and 45 PV)	97	2
	
	**CVMNK**	1 (PRG)	1.5	0
	
	C/S VMNT	1 (PV)	1.5	1 or 2

***pfdhfr***	ARICNVI	27 (14 PRG and 13 PV)	52	3
	
	ACICNVI	3 (1 PRG and 2 PV)	6	2
	
	ACICNVL	17 (1 PRG and 16 PV)	33	3
	
	ACNRNVI	1 (PV)	2	2
	
	ACIRNVI	1 (PV)	2	3
	
	ACNCNVI	1 (PRG)	2	1
	
	**ACNCSVI**	1 (PRG)	2	0
	
	ARICNV I/L	1 (PRG)	2	3 or 4

***pfdhps***	SGEGA	41 (17 PRG and 24 PV)	65	3
	
	SGEAA	9 (1 PRG and 8 PV)	14	2
	
	SGKAA	10 (PV)	16	1
	
	SGE A/G A	1 (PRG)	1.5	2 or 3
	
	SGE/K AA	1 (PV)	1.5	1 or 2
	
	SGE/K A/G A	1 (PV)	1.5	1, 2 or 3

Codon positions: *pfcrt *72 to 76 (n = 65); *pfdhfr *16, 50, 51, 59, 108, 140 and 164 (n = 52); and *pfdhps *436, 437, 540, 581 and 613 (n = 63). The sensitive haplotypes are shown in bold characters and the mutated codons are underlined.

A multilocus analysis among the 47 samples showing single haplotypes was performed. The *pfdhfr *+ *pfdhps *+ *pfcrt *genes presented some usual haplotype associations as, respectively, follows: ARICNVI + SGEGA + SVMNT (42.5%), ACICNVL + SGEGA + SVMNT (27.6%), ARICNVI + SGEAA + SVMNT (10.6%), ACICNVL + SGEAA + SVMNT (6.3%) and ACICNVI + SGEGA + SVMNT (4.2%). Other combinations such as ACICNVI + SGEAA + SVMNT (2.13%), ACNCNVI + SGEGA + SVMNT (2.13%), ACNCSVI + SGEGA + CVMNK (2.13%) and ACNRNVI + SGKAA + SVMNT (2.13%) were observed only once. In all these samples *pfdhfr *and *pfdhps *haplotypes were significantly associated (p < 0.0001, Chi-square test); the same was true for the only *P. falciparum *sample with CQ and SP sensitive genotypes.

The five amino acid insertion between codons 30 and 31 of the *pfdhfr *gene named "Bolivia repeat" was found in 17 samples (one from Paragominas and 16 from Porto Velho) and it was always associated with the leucine at the codon 164, as previously reported [17].

In the two studied localities, the prevalence of the haplotypes was different, for *pfdhfr*, *pfdhps *and *pfcrt *genes, respectively. In Paragominas the predominant haplotypes were ARICNVI, SGEGA and SVMNT while in Porto Velho, the main ones were ACICNVL, SGEGA and SVMNT.

## Discussion

Porto Velho displayed more allelic variation than Paragominas, especially for the *pfdhps *gene. No previous studies were performed with *P. falciparum *samples from Paragominas, but two former studies had been performed in Porto Velho using SP [2] or CQ [18] molecular markers. In these reports, the authors detected the haplotypes CVIET (*pfcrt *gene), AGEGA (*pfdhps *gene) and the S108T (*pfdhfr *gene) that were not seen in the present study. However, *pfdhps *and *pfdhfr *haplotypes such as SGEAA, SGKAA, ACIRNVI and ACNRNVI, were described for the first time in Brazil, while they had only previously been reported from Tanzania [15] and India [19]. This might be due to the considerable human movement in Porto Velho, not only from other Amazonian (SA) regions, but also from other areas in and outside Brazil, which could also explain the detection of 16 *P. falciparum *infections harbouring the "Bolivia repeat" against only one identified in Paragominas.

In Venezuela, a *pfcrt *wild type, similar to that found in Paragominas, was also detected [20]. Differently from Porto Velho, migration from the city of Paragominas to other SA countries is very uncommon and, therefore, the existence of a wild type parasite in Brazil seems not to be related to parasite migration from other SA countries. Since this infection harboured wild-type codons at *pfcrt *72, 74, and 75, it is less probable that this haplotype correspond to a single resistant allele that would have reverted at the critical 76 codon (back mutation) and, consequently, the more plausible explanation for this finding is the presence of a *bona fide *sensitive allele.

Interestingly, in this study, an inferior prevalence of mutated codons associated to SP resistance was perceived when compared *pfdhps *haplotypes with those already observed in Brazil [2]. For instance, in 1998, Porto Velho isolates displayed triple (53.4%) or quadruple (46.6%) mutants in this gene, while single (22.7%), double (18.2%) and triple (54.5%) mutants were detected in this study, but no quadruple mutation was identified.

The present study is the first to document the existence of CQ (CVMNK) and SP (ACNCSVI) sensitive haplotypes in a Brazilian field sample. This is a noteworthy result, because in Brazil the totally of *P. falciparum *parasites were considered to be fully resistant to CQ and SP [18]. This fact was not, however, surprising since the same phenomenon had already been reported in China and Malawi [9-11]. Then, it could represent the reemergence of CQ and SP sensitive parasites probably due to the spread of wild type allele *P. falciparum *parasites [21]. Despite the relatively small number of samples, it is suggested that the number of sensitive parasite population detected remains low because CQ is currently is still used for the treatment of malaria vivax cases and SP or its analogues, are used for anti-microbial therapy and, in this way, *Plasmodium *parasites could still be under drug pressure.

The finding of a single wild SP and CQ isolate will not interfere in drug policy, but this detection is especially remarkable because it might represent a tendency of sensitive haplotypes re-emergence that could gain some importance in the future.

## Conclusion

These data reinforce the importance of performing molecular surveillance by continuous chemoresistance assessment, not only to predict shifts of drug resistance of *Plasmodium *populations following drug policy changes but, particularly, to investigate the possibility of reintroduction of anti-malarial drugs, such as CQ and SP, because of their low cost and wide availability. In this context, the critical problem of *P. falciparum *chemoresistance could be circumvented by turning-over the use of the drugs, in order to enable the re-emergence of wild sensitive parasites.

## Abbreviations

*pfcrt*: *Plasmodium falciparum *chloroquine resistance transporter; *pfdhfr*: *Plasmodium falciparum *dihydrofolate reductase; *pfdhps*: *Plasmodium falciparum *dihydropteroate synthase; DNA: deoxyribonucleic acid; PCR: polymerase chain reaction; SNPs: single-nucleotide polymorphisms; CQ: chloroquine; SP: sulphadoxine-pyrimethamine.

## Competing interests

The authors declare that they have no competing interests.

## Authors' contributions

BEG participated in the design of the study, carried out the molecular analysis and drafted the manuscript; NKAO performed the PCR assays; MGZ performed the *pfcrt*-PCR standardization; JMS and FS helped in design of the study and field facilities for blood sample collections; CTDR helped in the design of the study and reviewed the manuscript; MFFC conceived the study, coordinated its design, and finalized the manuscript. All authors have read and approved the final text.
